# The Biochar Derived from Carp for High‐Efficiency Solar Steam Generation and Water Purification

**DOI:** 10.1002/gch2.202100083

**Published:** 2021-10-20

**Authors:** Hongtao Qiao, Baowei Zhao, Xidong Suo, Xiaoming Xie, Lifang Dang, Jie Yang, Bowen Zhang

**Affiliations:** ^1^ Department of Chemistry Xinzhou Teachers University 10 Heping West Street Xinzhou 034000 China; ^2^ School of Environmental and Municipal Engineering Lanzhou Jiaotong University Lanzhou 730000 China; ^3^ School of Electrical and Electronic Engineering Tiangong University Tianjin 300000 China

**Keywords:** meat biochar, solar thermal conversion, sunlight absorption, water purification

## Abstract

Efficient utilization of solar energy to generate steam is a green and promising technology because of its great potential applications in seawater desalination and industrial wastewater purification. However, the practical application of high‐efficiency solar steam generation devices is largely overshadowed due to their complex process, high cost, low life‐span, and poor thermal performance. Here, novel meat and bonemeal biochar (MBB) with high solar steam generation efficiency is produced by pyrolyzing dead carp at 300, 400, and 500 °C under anoxic conditions. Attributed to its typical hydrophilic pore structure, the photon trapping ability of MBB500 is up to 97% and 84.1% in the ultraviolet and visible regions and near‐infrared light regions, respectively. Meanwhile, hydrophilic pore structural provides a strong capillary force for the rapid transmission of water. As a result, under 1 sun illumination (1 kW m^−2^), the water evaporation rate and the apparent energy conversion efficiency of MBB500 reach 1.48 kg m^−2^ h^−1^ and 131.2%, respectively. In addition, MBB500 also exhibits excellent seawater and heavy metal wastewater evaporation effects, providing a new manufacturing strategy for photo‐thermal materials, which greatly benefit their practical application in pure water regeneration.

## Graphical abstract

Biochar prepared from dead carp is designed as a high efficient solar water evaporator. The pyrolysis temperature of biochar and solar light intensity have a significant impact on the evaporation efficiency of the biochar. Under 1 sun illumination, biochar prepared at 500 °C has a highest water evaporation rate (1.48 kg m^−2^ h^−1^) and an apparent energy conversion efficiency of 131.2%.

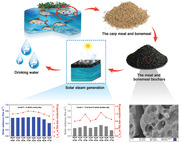

## 1 Introduction

The demand for fresh water is exponentially escalating due to the expansion of industrial activities, the rise in population rates, together with the enhanced living standards.^[^ ^1^ ^]^ However, available fresh water resources from rivers and groundwater account for less than 0.5% of the earth's total water supply, which seriously imperils the sustainability of society.^[^ ^2^ ^]^ Thus, the treatment of nonconventional water sources is necessary to ensure the sustainable water supply.^[^ ^3^ ^]^ With an average salinity of 35 000 mg L^−1^, seawater occupies about 97.5% of the earth's water, which represents the earth's major water reservoir.^[^ ^1^ ^]^ Recently, desalination technologies were employed to ensure a sustainable fresh water supply by removing salt from the virtually unlimited supply of seawater.^[^ ^3^, ^4^, ^5^ ^]^ Solar steam generation is emerging as one of the most promising seawater desalination and wastewater reuse technologies due to its high evaporation rate and excellent energy conversion efficiency.^[^ ^3^, ^4^, ^5^, ^6^, ^7^ ^]^ With clean solar irradiation as the only energy input, the high‐performance photothermal materials absorb and convert the energy of sunlight, forming the heat zones and generating the solar steam.^[^ ^8^, ^9^, ^10^ ^]^ Therefore, researchers are committed to develop high‐performance solar thermal conversion materials with high light absorption, low thermal conductivity, and excellent porosity.

The commonly used solar thermal conversion materials mainly include metallic plasmonic materials, semiconductor materials, and carbon‐based materials.^[^ ^11^, ^12^, ^13^, ^14^, ^15^, ^16^ ^]^ Zhou's team had successfully developed plasmon‐enhanced solar desalination devices, which were fabricated by self‐assembly of aluminum and gold nanoparticles into a 3D porous membrane.^[^ ^11^ ^]^ The results showed that the water evaporation rate with gold plasmonic absorber was 2.1 times of pure water at one sun solar irradiation (1 kW m^−2^), while the porous aluminum plasmonic absorber had effective desalination. For semiconductor materials, the water evaporation rate of Ti_2_O_3_ and black TiO_2_ under 1 kW m^−2^ solar irradiation was 1.32 and 1.13 kg m^−2^ h^−1^.^[^ ^17^ ^]^ The solar steam efficiency for Cu_7_S_5_ reached 81.3% and 91.3% under 2 and 2.5 kW m^−2^ irradiation, respectively.^[^ ^18^ ^]^ However, the instability of metal nanoparticles in corrosive media (such as acids, alkalis, and salts), and the narrow absorption band of semiconductor materials greatly limit their practical applications in seawater desalination and industrial wastewater treatment by solar steam generation.^[^ ^19^ ^]^ Carbon‐based materials have become one hotspot in the research of solar steam generation due to their broadband absorption characteristics and high chemical stability compared with metallic plasmonic and semiconductor materials.^[^ ^13^, ^20^ ^]^ However, due to the hydrophobicity of carbon based materials, especially graphene materials, surface modification must be performed to improve their wettability with water, because better wettability is beneficial to water transport and heat transfer.^[^ ^19^ ^]^ For example, vertically aligned graphene was prepared from natural graphite after freeze casting with freeze‐drying and thermal annealing, and the average water evaporation rate was 1.62 kg m^−2^ h^−1^ under 1 kW m^−2^ solar illumination.^[^ ^21^ ^]^ Yang et al. confirmed that the modification of graphene with hydrophilic groups greatly improve the efficiency of solar thermal steam generation from 38% to 48% under 1 kW m^−2^ solar irradiation.^[^ ^13^ ^]^ Although considerable progress have been made in carbon‐based materials, high‐cost preparation and modification processes still limit their practical applications.

As one of the carbon‐containing, stable, and highly aromatized carbonaceous solid material, biochar was prepared by pyrolyzing the pristine biomass materials under oxygen‐limited conditions and the temperature below 700 °C, which has received great attention in the field of environmental pollution remediation due to its high specific surface area, abundant surface functional groups (e.g., carboxyl, hydroxyl, and so on), and highly porous structure.^[^ ^22^, ^23^, ^24^, ^25^ ^]^ Due to the unique properties, simple design, and low cost, researches on biochar as the solar thermal conversion materials have gradually increased. The previous research demonstrated that biochars prepared from daikon,^[^ ^19^ ^]^ mushrooms,^[^ ^26^ ^]^ and *enteromorpha prolifera* ^[^ ^8^ ^]^ exhibited water evaporation rate of 1.57, 1.48, and 1.1–1.3 kg m^−2^ h^−1^, and solar steam efficiency of 85.9%, 78%, 80–84%, respectively, under 1 kW m^−2^ solar irradiation, which are much better than traditional thermal conversion materials. With the intensive aquaculture of aquatic products on a large scale, China has ranked first in both output and scale in the world. In intensive farming process, the inevitable large amount of byproducts, waste biomass including abnormally dead aquatic products, residual fishing bait and manure, etc., is burned or buried, which not only causes biomass energy waste and environmental pollution, but also cause the risk of human storage comorbidities. The pyrolysis of these waste biomass into biochar is used for solar steam generation, which will realize the reduction, harmlessness, and resource disposal of waste biomass resources in the aquaculture industry.

**Figure 1:**
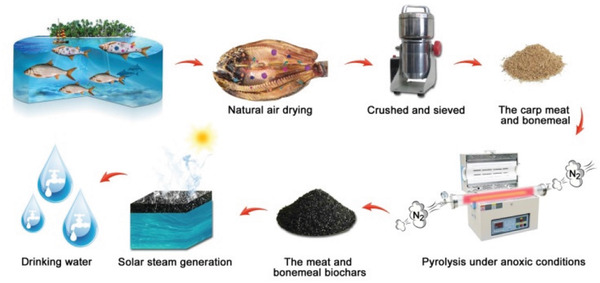
Schematic illustration depicting the preparation of the meat and bonemeal biochars (MBB) and setup for solar steam generation with MBB on water surface.

In this work, the meat and bonemeal biochars (MBB) were prepared at 300, 400, and 500 °C by pyrolysis of dead carp in anoxic condition as an effective solar thermal conversion material (**Figure** **1**). It was demonstrated that high energy conversion efficiency could be obtained in biochar prepared at 500 °C (MBB500). Due to the existence of developed pore structure and oxygen‐containing functional groups on the surface of MBB500, no additional hydrophilic modification is required, which greatly simplifies the preparation process. The pore structures not only serve as traps for photons capturing to improve the solar absorption over 86.4% within the main solar spectrum, but also provide strong capillary force for the rapid transmission of water. Under one sun illumination (1 kW m^−2^), MBB500 exhibited better water evaporation rate of 1.48 kg m^−2^ h^−1^ and the apparent energy conversion efficiency of 131.2%. In addition, almost no changes could be observed in energy conversion efficiency after ten‐cycles test. The outstanding performance of MBB500 confirmed their potential application in desalination process and heavy metal wastewater treatment. The introduction of MBB as solar thermal conversion materials not only provides new strategy for the reduction and recycling of waste biomass, but also proposed new pathway for the design of next generation solar thermal conversion materials.

## 2 Results and Discussion

**Figure 2:**
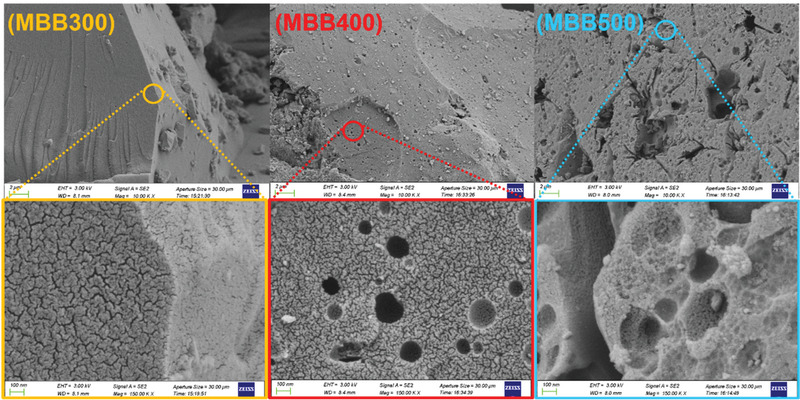
SEM images of MBB300, MBB400, and MBB500. The first line is the result of MBB amplification of 10 000 times, and the second line is the result of MBB amplification of 150 000 times.

**Figure 3:**
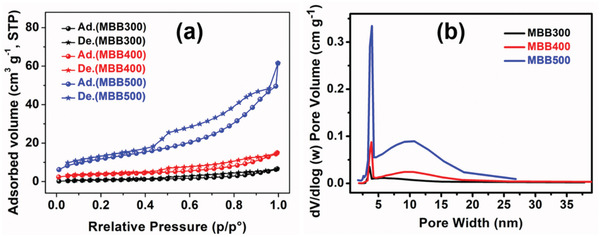
BET characterization results of biochar MBB300, MBB400, and MBB500. a) N2 adsorption–desorption curves of MBB300, MBB400, and MBB500; b) Pore size distribution curves of MBB300, MBB400, and MBB500.

The type of biomass and pyrolysis temperature are the main factors affecting the physical and chemical properties of biochars.^[^ ^28^, ^29^, ^30^ ^]^ The microstructure of the biochar prepared at 300, 400, and 500 °C were obtained as depicting in **Figure** **2**. It could be obviously observed that the surface roughness and surface cracks of MBB gradually increases with the increase of pyrolysis temperature, which mainly due to the slow combustion of organic matter and accumulation of minerals during biomass pyrolysis, such as bone, fat, and connective tissue in the carp meat and bonemeal.^[^ ^27^, ^28^ ^]^ The surface morphology of MBB and lignocellulosic biochars (such as rice straw biochar,^[^ ^29^ ^]^ peanut shell biochar and Chinese medicinal residue biochar^[^ ^30^ ^]^) are completely different. There is no regular honeycomb structure on MBB surface, but there are irregular cracks, which is mainly caused by the different composition of carp meat and bonemeal and lignocellulose. The cracks produced during the pyrolysis process were likely to act as photon captured traps to increase solar energy absorption, and act as microchannels to support sufficient water transport and vapor escape at the same time.^[^ ^19^, ^26^ ^]^ The Brunauer–Emmett–Teller (BET) characterization results showed that (**Figure** **3**), as the pyrolysis temperature increased from 300 to 500 °C, the specific surface area of biochars increased from 3.1 to 52.3 m^2^ g^−1^, and the pore volume increased from 0.01 to 0.08 cm^3^ g^−1^, which further confirm the microstructure evolution shown in Figure 2. The higher specific surface area could increase the contact area between biochar and water, thereby increasing the heat transfer from the heated biochar to the water, and increasing the efficiency of solar steam.

**Figure 4:**
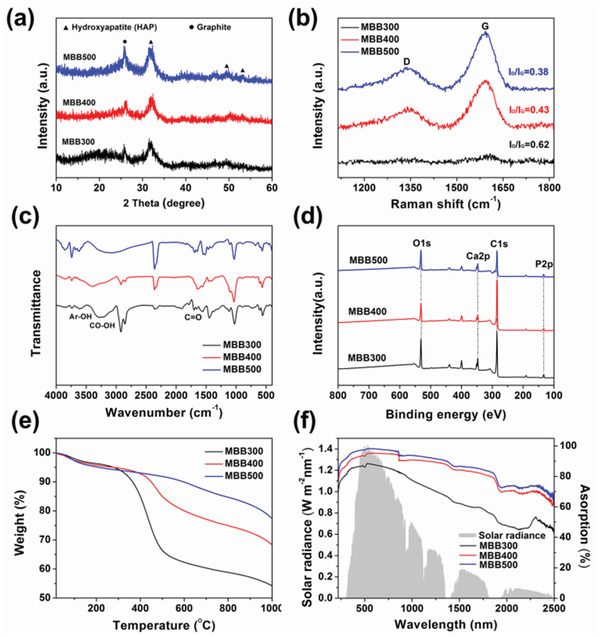
a) XRD pattern and b) Raman spectrum of MBB. c) XPS spectra of survey scan and d) FTIR spectra for MBB. e) Thermogravimetric curves of MBB. f) Solar spectral irradiance (AM 1.5 G) (left axis) and absorption (right axis) of MBB with different pyrolysis temperatures.

The XRD pattern of MBB300, MBB400, and MBB500 was shown in **Figure** **4**a to investigate their phase changes. The results showed that MBB contained hydroxyapatite [HAP, Ca_10_(PO_4_)_6_(OH)_2_],^[^ ^27^, ^28^ ^]^ which was mainly derived from fish bones. As seen from Figure S3, Supporting Information, the SEM‐EDS results (Figure S3, Supporting Information) could also provide direct evidence. The distribution of Ca—P—O in MBB surface exhibited a notable correlation in the elemental dot maps of EDX spectra, which cannot be affected by the increase of pyrolysis temperature. This was consistent with the previous results that a large amount of HAP existed in animal‐derived biochar.^[^ ^27^ ^]^ In addition, the peaks around 26° was the typical reflection of graphite, which corresponded to the representative (002) diffractions of graphite, indicating that the sample contained graphene like structure.^[^ ^19^, ^31^ ^]^ The degrees of graphitization of MBB were further characterized by Raman spectroscopy (Figure 4b). As shown in Figure 4b, two strong peaks were observed at ≈1340 and ≈1590 cm^−1^, which represented the disorder D‐band of amorphous carbon and the in‐plane sp^2^ vibration of graphite crystal G‐band, respectively.^[^ ^19^ ^]^ The degree of graphitization of carbonaceous materials could generally be estimated from the intensity ratio of the D peak to the G peak (*I*_D_/*I*_G_). As the pyrolysis temperature increased from 300 to 500 °C, the *I*_D_/*I*_G_ value of biochar decreased from 0.62 to 0.38, indicating that the preparation temperature was beneficial to the graphitization degree of MBB and negative to the content of amorphous carbon.

The surface functional groups and chemical compositions of MBB were analyzed by Fourier transform infrared spectroscopy (FTIR) and X‐ray photoelectron spectroscopy (XPS). As shown in Figure 4c, the representative signals of the PO_4_^3−^ were recorded at 1029, 563, and 468 cm^−1^, and the bands in 875 cm^−1^ were attributed to CO_3_^2−^.^[^ ^19^, ^23^, ^27^, ^29^ ^]^ The vibrations of Ar—OH/CO—OH and C=O were observed at 3200–3700 and 1710 cm^−1^, respectively.^[^ ^19^, ^23^, ^27^ ^]^ The XPS survey spectrum (Figure 4d) showed that MBB prepared at different pyrolysis temperatures were mainly composed of four elements, including carbon (70–82%), oxygen (13–22%), calcium (2–5%), and phosphorus (2–4%). The high resolution spectrum for the C1s peak can be deconvolved into three components including aliphatic C=C, hydroxyl carbon C—O, and carbonyl carbon C=O (Figure S4, Supporting Information). The presence of several oxygen‐containing groups such as C=O and C—O were also confirmed by the deconvolved O1s spectrum (Figure S4, Supporting Information). In addition, the water contact angle (Figure S5, Supporting Information) was estimated to be 75.2–82.3^o^ (less than the critical angle of 90^o^), which demonstrated the hydrophilic surface of MBB. This hydrophilic property could be attributed to the hydrophilic oxygen‐containing functional groups, such as —C=O, —C—O, and —COOH. The combination of hydrophilicity and well‐developed pore structure will produce excellent capillary action, realizing the rapid transportation and distribution of water, which was beneficial to the generation of solar steam.

To improve the solar thermal conversion efficiency, thermal stability and the sunlight utilization rate of the solar thermal conversion materials should be focused on. The thermal stability of MBB was investigated by thermogravimetry (Figure 4e). The mass loss of water evaporation and structure‐bound water (30–200 °C) was basically about 4% for MBB. However, the mass loss of organic matter (200–600 °C).^[^ ^32^ ^]^ significantly decreased from 62.3% in MBB300 to 9.6% in MBB500 (18.6% in MBB400). Relative to the MBB300 and MBB400, MBB500 consisted of a more stable form of carbon and thus had a high thermal stability. In addition, the solar energy absorption of MBB was carefully measured with an ultraviolet visible near infrared spectrophotometer equipped with an integrating sphere, and compared to the solar radiation spectrum (250–2500 nm),^[^ ^33^, ^34^, ^35^ ^]^ as shown in Figure 4f. With the pyrolysis temperature increased from 300 to 500 °C, the overall solar energy absorption of MBB increased from 66.9 to 86.4%, the absorption in the ultraviolet and visible regions (300–780 nm) increased from 86.5% to 97%, and the absorption in the near infrared region (780–2500 nm) increased from 62.1% to 84.1%. The inorganic salt impurities contained in MBB, such as HAP, carbonate and phosphate (Figure 4a,c), may be one of the reasons why the overall solar energy absorption of MBB is difficult to reach ≈100%.^[^ ^8^ ^]^ With the increase of pyrolysis temperature, the absorption of MBB in the whole wavelength range, the ultraviolet and visible wavelength regions and the near infrared wavelength region has shown an increasing trend, which is mainly due to three reasons: the first is the increase of specific surface area of MBB (Figure 3), resulting in the increase of contact area between material and sunlight;^[^ ^35^, ^36^, ^37^ ^]^ Second, MBB prepared at high temperature has more porous structure (Figure 2), which increases the optical path and reduces the light reflection;^[^ ^36^ ^]^ third, the graphitization degree of MBB increases with the increase of pyrolysis temperature, which enhances the absorption of MBB in the near‐infrared wavelength range.^[^ ^37^ ^]^ The evolution of energy absorption was consistent with the characterization results of SEM and BET, which confirms that the high surface roughness and cracks of MBB enable strong and broad optical resonances, thus producing efficient light trapping and absorption enhancement.^[^ ^33^, ^34^, ^35^, ^36^, ^37^ ^]^ In addition, the saturated water content of MBB was tested. The results showed that the saturated water content of MBB300, MBB400, and MBB500 were 1.58, 1.65, and 2.01 g g^−1^ respectively, which was mainly because the specific surface area and the pore volume of MBB increased with the increase of pyrolysis temperature, resulting in the maximum saturated water content of MBB500 to ensure that it is able to absorb the water needed for vaporization.

**Figure 5:**
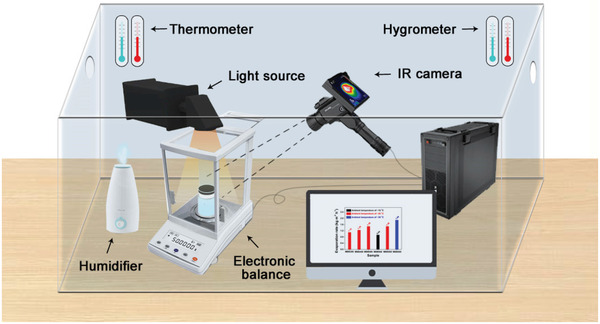
The schematic diagram of the entire device for solar evaporation experiments.

**Figure 6:**
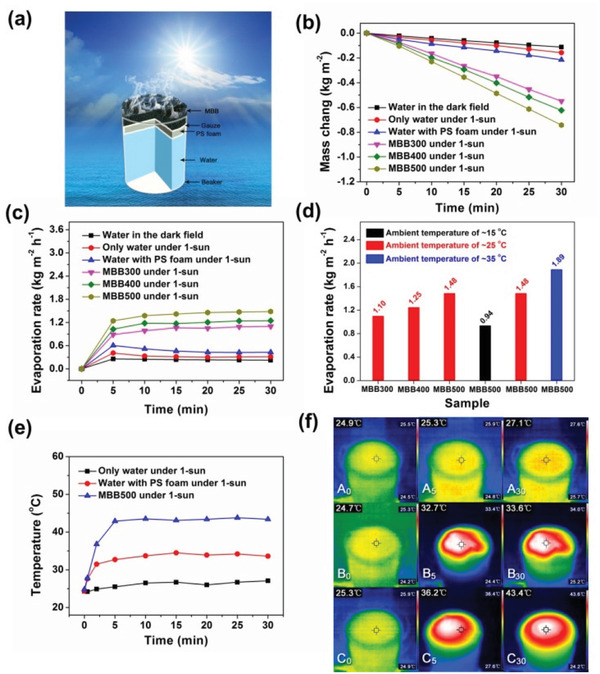
a) Schematic illustration of solar steam generation. b) Vapor evaporation induced mass changes of water as a function of time in the dark and under one sun illumination. c) Water evaporation rate comparison in the dark and under one sun illumination. d) Water evaporation rate comparison of MBB300, MBB400, and MBB500 at different ambient temperatures. e) The temperature variation at MBB500 surface and water surface with/without PS foam. f) Infrared photos of temperature distribution under one sun illumination: A0, A5, and A30 respectively indicated the temperature distribution of only water surface at 0, 5, and 30 min (the first row); B0, B5, and B30 respectively indicated the temperature distribution of water surface with PS foam at 0, 5, and 30 min (the second row); C0, C5, and C30 respectively indicated the temperature distribution of MBB500 surface at 0, 5, and 30 min (the third row).

In order to evaluate the water evaporation rate of MBB under one sun illumination, steam generation measurements were performed by an artificial optical system at an ambient temperature of ≈25 °C and a humidity of 41%. The schematic diagram of the entire device for solar evaporation experiments and the schematic illustration of solar steam generation were shown in **Figures** **5** and **6**a, respectively. As illustrated in inset image of Figure 6a, PS foam with a thickness of 1 cm could act as a thermal barrier to realize localized heating and inhibit heat conduction down to bulk water. The PS foam was wrapped with of cotton cloth to enable efficient water supply, and each MBB was directly placed on it. Figure 6b showed the mass change during the transportation process of water at different conditions. Under one sun illumination, the water mass changes of MBB300, MBB400, and MBB500 within 30 min were 0.55, 0.62, and 0.74 kg m^−2^, respectively, which corresponding to 3.5, 3.9, and 4.7 times of pure water. Due to the low thermal conductivity of gauze wrapped PS foam (0.0485 W m^−1^ K^−1^), the water mass changes was only 1.3 times to pure water under one solar radiation. The results of the water evaporation rate over time were shown in Figure 6c. For The evaporation rates presented three stages: rapid increase in 0–5 min, slow increase in 5–20 min and gradual stability after 20 min. The direct comparison in Figure 6d showed that MBB500 exhibited the highest water evaporation rate under one sun illumination, which was 1.3 and 1.2 times to MBB300 and MBB400, respectively. The evaporation performance of MBB had a positive correlation with the pyrolysis temperature. With the increase of ambient temperature from 15 to 35 °C, the evaporation rate of MBB500 significant increased from 0.94 to 1.89 kg m^−2^ h^−1^, indicating that the ambient temperature was one of the important factors affecting the evaporation rates.

In order to better understand MBB500's high efficiency solar driven water evaporation mechanism, the temperature variation (Figure 6e) and temperature distribution (Figure 6f) were recorded by an infrared camera at an ambient temperature of ≈25 °C and a humidity of 41%. When MBB500 was placed on the gauze wrapped PS foam for solar driven water evaporation, the surface temperature of MBB500 increased rapidly from 24.8 to 42.9 °C within 5 min, and then slowly increased to 43.4 °C and gradually stabilized after 15 min. The surface temperature increase of MBB500 (18.6 °C) was significantly higher than that of pure water (2.8 °C) and the gauze wrapped PS foam (9.2 °C). Combined with the data of temperature distribution (Figure 6f), it can be seen that CMBB500 had achieved local heating, which improved the solar evaporation rate.

The performance of the MBB based solar thermal evaporator was evaluated by the photothermal conversion efficiency, and the equation was shown below^[^ ^34^, ^38^ ^]^

(1) $$\begin{array}{c} \eta =mh_{\text{LV}}/C_{\text{opt}}q_{\text{in}} \end{array}$$

(2) $$\begin{array}{c} h_{\text{LV}}=\lambda_{\text{LV}}+C_{P}\Delta T \end{array}$$

where η is the photothermal conversion efficiency; *m* is the net evaporation rate of steam generation, which is the difference between the net water under sunlight and under darkness (pure water evaporation rate of 0.22 kg m^−2^ h^−1^); *C*_opt_ and *q*_in_ are the optical concentration and the solar irradiation on the surface, respectively; *h*_LV_ represents liquid vapor phase change enthalpy; λ_LV_ and *C*_p_ denote the latent heat of water evaporation under the standard atmospheric pressure (2.257 MJ kg^−1^) and the specific heat capacity of bulk water (4.2 kJ kg^−1^ K^−1^); Δ*T* represents the variation of temperature in the evaporation process. The apparent conversion efficiency of MBB500 was 131.2% at an ambient temperature of ≈25 °C and a humidity of 41%, which was significantly higher than MBB300 (71.1%) and MBB400 (95.5%). Compared with MBB300 and MBB400, MBB500 had a wide and high absorption rate of sunlight (Figure 4f) and a well‐developed hydrophilic pore structure on the surface (Figure 3), which ensures that it had a high apparent efficiency based on the high utilization of sunlight and continuous water transmission to yield fresh water. Compared with other materials shown in Table S1, Supporting Information MBB500 showed the highest evaporation rate among graphite materials, the third highest evaporation rate among all materials, and the highest apparent conversion efficiency among all materials. Nevertheless, CMBB500 has significant advantages in terms of lower cost, simpler preparation process, environment‐friendly, and high‐stability.

It should be noted that the apparent conversion efficiency of MBB500 exceeded 100%, reaching 131.2%, which seems contrary to the regulation of energy conservation. This may be due to the occurring energy recovery mechanism in a relatively closed environment.^[^ ^38^, ^39^, ^40^ ^]^ The solar driven water evaporation process of MBB500 was taken place on an electronic balance, which was covered by glass plates to create a relatively closed environment inside. The whole system, including electronic balance, artificial light source, computer, etc., was in an acrylic box with stable temperature (≈25 °C) and humidity (41%) (Figure 5). However, when MBB500 was exposed to the simulated sunlight, the internal space of the electronic balance receives energy generated by the diffuse reflected light and irradiation, causing the significant increase of temperature in the internal space of the electronic balance over 25 °C in the acrylic box, with a temperature gradient of about 5 °C. As the internal temperature of the electronic balance rose, this energy would in turn reheat the water, increasing the water temperature and accelerating the evaporation of water, so that the apparent conversion efficiency of MBB500 was higher than 100%. Furthermore, the inhomogeneity of MBB500 interface temperature would lead to some spatial difference in latent and sensible heat, and the energy efficiency is calculated based on the spatial average temperature of the nonuniform surface.^[^ ^36^ ^]^ Therefore, this calculation may lead to overestimation of energy efficiency. The same situation also appeared in Zeng's research.^[^ ^36^ ^]^ Their research results suggested that the high conversion efficiency was only based on the calculation formula, even if exceeded 100%, it could still be used to compare the conversion rates of different photothermal materials.

**Figure 7:**
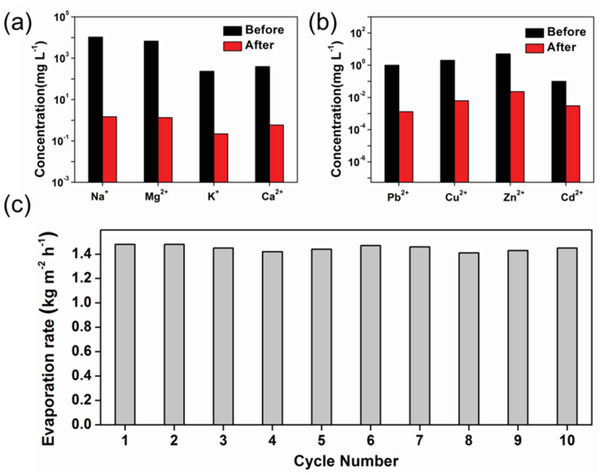
Concentrations of a) Na+, Mg2+, K+, Ca2+ in the seawater and b) Pb2+, Cu2+, Zn2+, Cd2+ in heavy metal wastewater before and after solar thermal desalination. c) The evaporation rate cycle performance of MBB500 under one sun illumination.

In order to characterize the potential application of MBB500 in seawater desalination and heavy metal wastewater purification, Na^+^, Mg^2+^, K^+^, and Ca^2+^ were selected to simulate the configuration of sea water, and Pb^2+^, Cu^2+^, Zn^2+^, and Cd^2+^ were selected to simulate the configuration of heavy metal wastewater.^[^ ^41^ ^]^ As shown in **Figure** **7**a, after the desalination process of MBB500, the concentration of Na^+^, Mg^2+^, K^+^, and Ca^2+^ were reduced from the initial 10534, 6720, 234, 396 mg L^−1^ (much higher than the World Health Organization drinking water standards of 50, 100, 10, and 100 mg L^−1^) to 1.5, 1.3, 0.2, 0.6 mg L^−1^ (lower than the World Health Organization drinking water standards), respectively.^[^ ^19^, ^38^ ^]^ Similarly, the initial concentrations of Pb^2+^, Cu^2+^, Zn^2+^, and Cd^2+^ were 1, 2, 5, 0.1 mg L^−1^ respectively (Figure 7b). After MBB500 purification, the concentration of Pb^2+^, Cu^2+^, Zn^2+^, and Cd^2+^ were reduced by about three orders of magnitude, reaching 0.001, 0.006, 0.023, 0.003 mg L^−1^, respectively, which fully meets China's drinking water standards of 0.01, 1.0, 1.0, 0.005 mg L^−1^ (Chinese National Standard GB5749‐2006).^[^ ^38^ ^]^ MBB500 was found to be stable water generation performance and could be reused multiple times (over ten cycles) without any noticeable degradation in the solar steam efficiency (Figure 7c). The cycle error of the efficiency was within ±2.4%. In addition, the evaporation performance of MBB500 in different outdoor weather conditions has been studied. The results show that the water evaporation rate of MBB500 is between ≈0.79–2.01 kg m^−2^ h^−1^ on sunny days and between ≈0.80–1.27 kg m^−2^ h^−1^ on broken sky from 9:30 a.m. to 17:30 p.m. Although the outdoor light intensity is less 1 kW m^−2^, the evaporation rate of MBB500 is higher than that in a relatively stable environment in the laboratory due to a certain intensity of wind outdoors. The evaporation rate on sunny days is significantly higher than that on broken sky, indicating that the light intensity is also an important factor. In summary, these results showed that MBB500 was a stable, efficient, and reusable solar thermal conversion material, which could be used in seawater desalination and heavy metal wastewater purification.

## 3 Conclusion

In this study, high solar driven water evaporation performance was achieved by the MBB that derived from the pyrolysis of carp meat and bonemeal at 500 °C under oxygen‐limited conditions. The efficiency of solar absorption was reached up to 86.4% for MBB500 owing to the well‐developed pore structure, hydrophilic interface, and high water transmission effect from the bottom to the biochar surface. Meanwhile, the water evaporation rate of MBB500 can reach up to 1.48 kg m^−2^ h^−1^ with apparent energy conversion efficiency of 131.2% at an ambient temperature of ≈25 °C and a humidity of 41%. Moreover, seawater and heavy metal wastewater purified by MBB500 can meet the standard of drinking water. In short, MBB500 shows great performance and promising potential application in the purification of seawater heavy metal wastewater. This study not only proves that carbonized biomass is a promising method for photothermal conversion, but also provides a new way for waste biomass utilization in the aquaculture industry.

## 4 Experimental Section

### Preparation of Meat and Bonemeal Biochar

The carp meat and bonemeal was prepared by crushing the carp meat into small pieces and passing through a 0.84 mm sieve (20 mesh), then the carp meat and bonemeal was dried in an oven at 70 °C for 24 h. After that, the dried carp meat and bonemeal was tightly placed in a ceramic pot, and then pyrolyzed in tube furnace with nitrogen atmosphere. The heating rate was 15 °C min^−1^, and the peak temperature (300, 400, and 500 °C) was maintained for 6 h before cooling to room temperature. All the biochar samples were passed through a 0.17 mm sieve (80 mesh), and then stirred with cyclohexane solution overnight (1:40 w/v) to remove the bio‐oil attached to the surface of the biochar. After that, the sand core funnel was used for suction filtration and ethanol was used for washing until the filtrate was clear. Compared with the filtrate, the content of bio‐oil gradually decreased with the increase of pyrolysis temperature (Figure S1, Supporting Information). All the biochar samples on the filter cake was baked in the oven at 85 °C for 4 h to obtain the MBB prepared at different pyrolysis temperatures, which were labeled MBB300, MBB400, and MBB500 in accordance with the pyrolysis temperature, respectively.

### Characterization

The pore volume, BET surface area and pore size of biochars were obtained by using a surface area and porosimetry analyzer (micromeritics, ASAP2020C, USA) via N_2_ adsorption tests. Thermal analysis of biochars were obtained by using a thermogravimetric analyzer (STA449F5, Netzsch, Germany) with the temperature risen from 30 to 1000 °C by a heating rate of 10 °C min^−1^ under an atmosphere of N_2_. The surface structure and elemental composition of biochars were visualized by using a JSM‐7800F SEM with EDS (Japan). The surface chemical composition was determined by X‐ray diffraction (Haoyuan, DX2700B, China) and X‐ray photoelectron spectroscopy (Thermo Scientific, ESCALAB 250Xi, USA). The surface functional groups of biochars were identified by FTIR (Bruker, TENSOR 27, Germany) between wave numbers 4000 to 400 cm^−1^. Raman spectra were recorded with a dispersive Raman microscope (Horiba Jobin Yvon LabRAM HR800, France) at an excitation wavelength of 532 nm. The water contact angle of biochars was measured using a contact angle meter (KRUSS DSA100, Germany) at ambient temperature using a 10 mL droplet as the indicator. Absorption spectra were measured on a UV–visible spectrometer over a range of 300–2500 nm (PerkinElmer, Lambda 750S, USA). The concentrations of Na^+^, Mg^2+^, K^+^, Ca^2+^, Cu^2+^, Cd^2+^, Pb^2+^, and Zn^2+^ were measured by inductively coupled plasma emission spectrometer (Optima 8000, PerkinElmer, USA).

### Experimental Procedure for Solar Steam Generation

MBB prepared at different pyrolysis temperatures was put on a hydrophobic polystyrene foam (a low thermal conductivity of ≈0.04 W m k^−1^), which was wrapped with cotton cloth. The whole structure was allowed to float on the water surface, only the cotton cloth at the bottom of the polystyrene foam was in direct contact with bulk water. The water evaporation experiments were carried out at ≈15, ≈25, ≈35 °C and a humidity of 41% using an artificial optical system. The digital images of the device were shown in Figure S2, Supporting Information. A xenon lamp (PLS‐SXE300, Perfect light, China) with a filter (AM 1.5) was used to simulate the solar sunlight. The light intensity was calibrated with a light intensity meter (FZ‐A, Beijing normal university, China). In a solar (AM 1.5G, 1 kW m^−2^) and dark condition, the changes of water mass and temperature with time were measured for 30 min at steady‐state condition by a high‐precision electronic balance connected to a computer and an infrared camera (ST9450, SMART SENSOR, China), respectively, and the evaporation rate and efficiency of solar steam generation was calculated based on this.

## Conflict of Interest

The authors declare no conflict of interest.

## Supporting information

Supporting Information

## Data Availability

Research data are not shared.
