# Supplementary material for: The Biochar Derived from Carp for High‐Efficiency Solar Steam Generation and Water Purification
Source: Glob Chall. 2021 Oct 20;6(1):2100083. doi: 10.1002/gch2.202100083 (PMC8727727; doi:10.1002/gch2.202100083)
Supplement: Supplementary file 1 — Supporting Information [file GCH2-6-2100083-s001.pdf]

Global Challenges
Open Access

Supporting Information

for *Global Challenges,* DOI: 10.1002/gch2.202100083

The Biochar Derived from Carp for High-Efficiency Solar Steam Generation and Water Purification

Hongtao Qiao, Baowei Zhao, Xidong Suo, Xiaoming Xie, Lifang Dang, Jie Yang,* and Bowen Zhang**

Supporting Information

The biochar derived from carp for high-efficiency solar steam generation and water purification

The biochar derived from carp for high -efficiency solar steam generation and water purification

Hongtao Qiao[a], Baowei Zhao[b], Xidong Suo[*a], Xiaoming Xie[a], Lifang Dang[a], Jie Yang[*a], Bowen Zhang[*c]

[a] Department of Chemistry, Xinzhou Teachers University, 10 Heping West Street, Xinzhou, China

[b] School of Environmental and Municipal Engineering, Lanzhou Jiaotong University, Lanzhou China.

[c] School of electrical and electronic engineering, Tiangong University, Tianjin, China

Correspondence to: Xidong Suo (E -mail: *xidsuo@126.com*); Jie Yang (E -mail: 545431607@qq.com); Bowen Zhang (bowenzhang192@gmail.com).

Table S1 The water evaporation rates and conversion efficiency of different materials under one sun illumination.

Materials	T^{α}/^{o}C	H^{α}/%	v^{β}/kg·m^{-2}·h^{-1}	η^{β}(%)	References
Carbonized daikon	~28	NO$^{\gamma}$	1.57	85.9	[1]
Carbonized mushrooms	~28	41	1.475	78	[2]
Carbonized enteromorpha prolifera	~22	35	1.1-1.3	80-84	[3]
Carbonized wood slice	~30	60	1.45	91.3	[4]
Carbonized melamine foams	~27	50	1.27	87.3	[5]
Hollow carbon spheres	~25	50	1.24-1.45	NO$^{\gamma}$	[6]
Carbonized carrot	~25	41	2.04	127.8	[7]
Commercially available activated carbon fiber felt	~25	NO$^{\gamma}$	1.22	79.4	[8]
Carbon black nanoparticles	~24	35	1.47	100	[9]
Graphite-coated wood	~21	NO$^{\gamma}$	1.15	80	[10]
PTC gel	NO$^{\gamma}$	NO$^{\gamma}$	1.49	93.8	[11]
3D hydrogel evaporators	NO$^{\gamma}$	NO$^{\gamma}$	1.42	96	[12]
the meat and bonemeal biochars	~25	41	1.48	131.2	This work

$^{\alpha}$ *T* and *H* represent the ambient temperature and humidity of the experimental process of solar steam generation, respectively.

$^{\beta}$ *v* and *η* represent the water evaporation rates and conversion efficiency, respectively.

$^{\gamma}$ “NO” represents the value of the corresponding item not mentioned in the references.

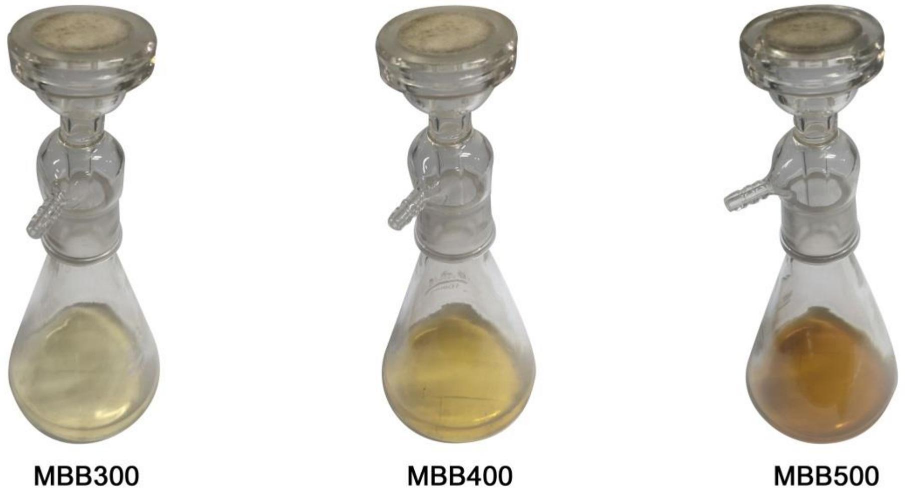

Figure S1 Cleaning filtrates of MBB300, MBB400 and MBB500 with cyclohexane and ethanol.

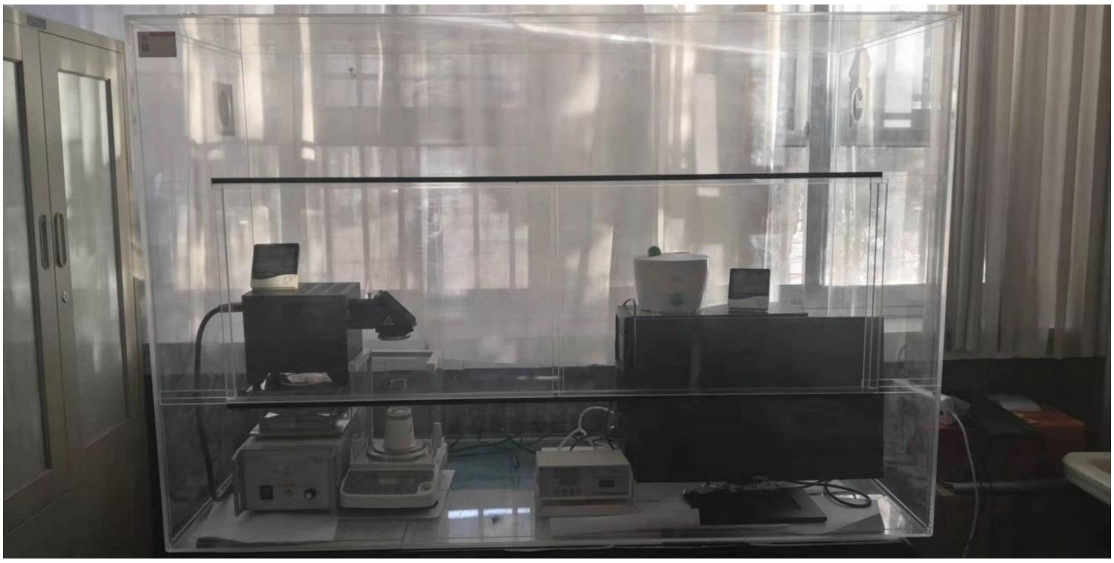

Figure S2 Digital images of experimental device.

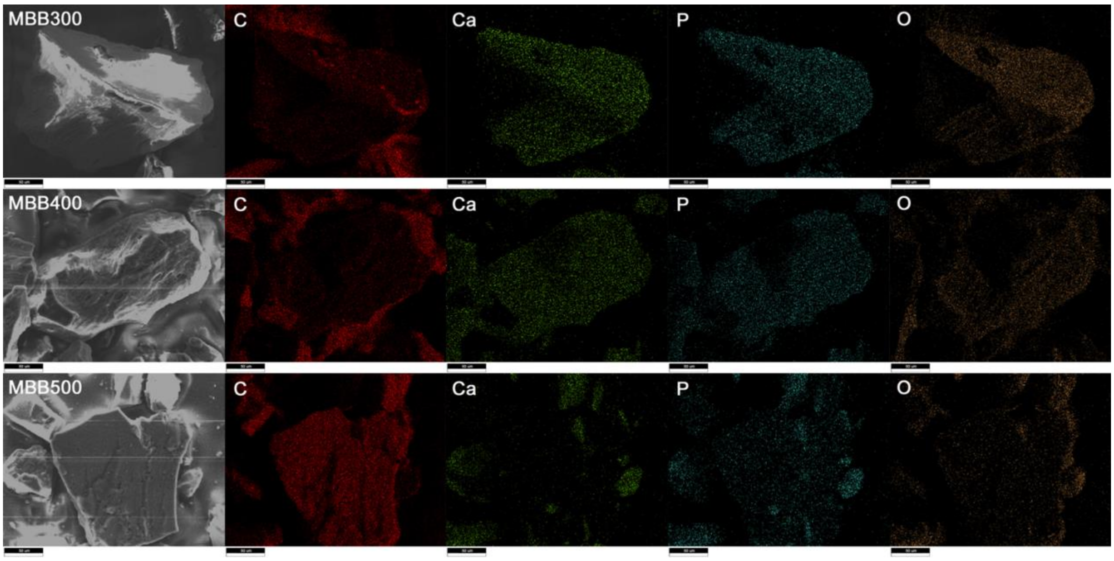

Figure S3 SEM images and elemental dot maps of MBB300, MBB400 and MBB500.

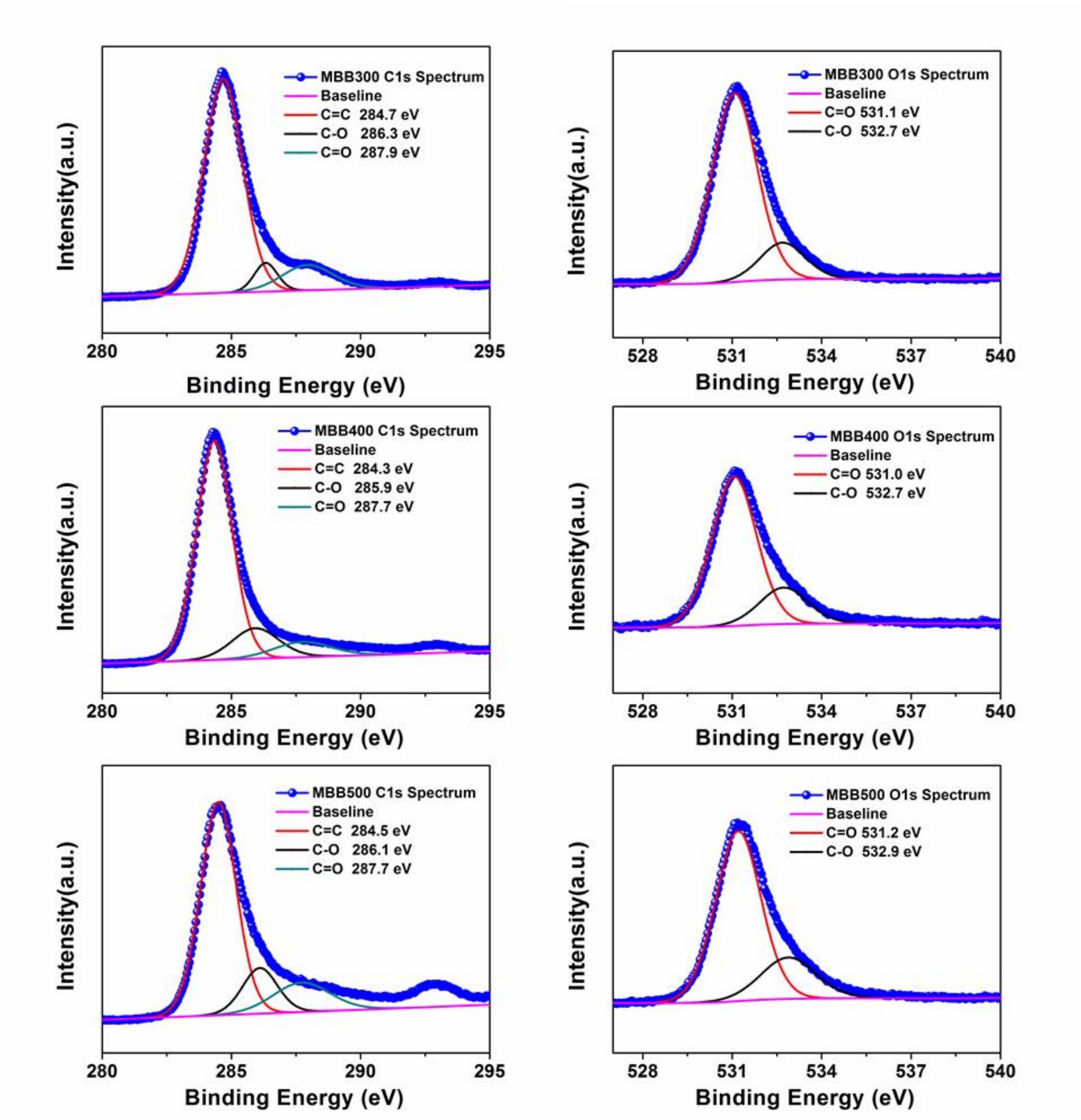

Figure S4 High resolution XPS with shirely fitting analysis of C1s and O1s for MBB300, MBB400 and MBB500.

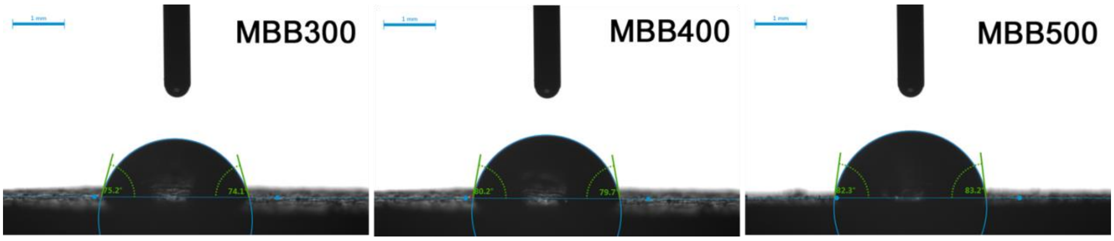

Figure S5 Water contact angle of MBB300, MBB400, and MBB500.

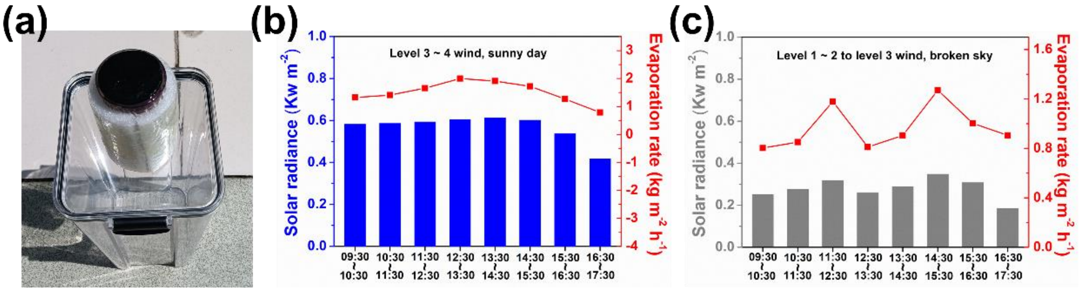

Figure S6 (a) Outdoor experiment pictures; (b-c) The solar intensity and the water evaporation rate of MBB500 at different time periods in outdoor evaporation experiment.

References:

[1] M. Zhu, J. Yu, C. Ma, C. Zhang, D. Wu, H. Zhu, Solar Energy Materials and Solar Cells, **2019**, 191, 83-90.

[2] N. Xu, X. Hu, W. Xu, X. Li, L. Zhou, S. Zhu, J. Zhu, Advanced Materials, **2017**, 29(28), 1606762.

[3] L. Yang, G. Chen, N. Zhang, Y. Xu, X. Xu, ACS Sustainable Chemistry & Engineering, **2019**, 7(23), 19311-19320.

[4] P. F. Liu, L. Miao, Z. Deng, J. Zhou, H. Su, L. Sun, S. Tanemura, W. Cao, F. Jiang, L. D. Zhao, Materials Today Energy, **2018**, 8, 166-173.

[5] X. Lin, J. Chen, Z. Yuan, M. Yang, G. Chen, D. Yu, M. Zhang, W. Hong, X. Chen, I Journal of Materials Chemistry A, **2018**, 6(11), 4642-4648.

[6] J. Zhou, Z. Sun, M. Chen, J. Wang, W. Qiao, D. Long, L. Ling, Advanced Functional Materials, **2016**, 26(29), 5368-5375.

[7] Y. Long, S. Huang, H. Yi, J. Chen, J. Wu, Q. Liao, H. Liang, H. Cui, S. Ruan, Y. J. Zeng, Journal of Materials Chemistry A, **2019**, 7(47), 26911-26916.

[8] H. Li, Y. He, Y. Hu, X. Wang, ACS Appl Mater Interfaces, **2018**, 10(11), 9362-9368.

[9] X. Li, J. Li, J. Lu, N. Xu, C. Chen, X. Min, B. Zhu, H. Li, L. Zhou, S. Zhu, T. Zhang, J. Zhu, Joule, **2018**, 2(7), 1331-1338.

[10] T. Li, H. Liu, X. Zhao, G. Chen, J. Dai, G. Pastel, C. Jia, C. Chen, E. Hitz, D. Siddhartha, R. Yang, L. Hu, Advanced Functional Materials, **2018**, 28(16), 1707134.

[11] M. Gao, C. K. Peh, L. Zhu, G. Yilmaz, G. W. Ho, Adv. Energy Mater. 2020, 10, 2000925.

[12] Y. Zhou, T. Ding, M. Gao, K. H. Chan, Y. Cheng, J. He, G. W. Ho, Nano Energy, 2020, 77, 105102.
